# In Vitro Acaricidal Activity of Silver Nanoparticles (AgNPs) against the Poultry Red Mite (*Dermanyssus gallinae*)

**DOI:** 10.3390/pharmaceutics15020659

**Published:** 2023-02-16

**Authors:** Georgios Sioutas, Alexandros Tsouknidas, Athanasios I. Gelasakis, Afrodite Vlachou, Alexandra K. Kaldeli, Maria Kouki, Isaia Symeonidou, Elias Papadopoulos

**Affiliations:** 1 Laboratory of Parasitology and Parasitic Diseases, Faculty of Health Sciences, School of Veterinary Medicine, Aristotle University of Thessaloniki, 54124 Thessaloniki, Greece; 2 PLiN Nanotechnology S.A., Spectra Business Center 12th km Thessaloniki-Chalkidiki, Thermi, 57001 Thessaloniki, Greece; 3 Laboratory of Anatomy and Physiology of Farm Animals, Department of Animal Science, School of Animal Biosciences, Agricultural University of Athens, 11855 Athens, Greece

**Keywords:** *Dermanyssus gallinae*, silver nanoparticles, AgNPs, scanning electron microscopy, bioassays, poultry red mite, PRM, contact toxicity

## Abstract

*Dermanyssus gallinae* (PRM) is the most common blood-sucking ectoparasite in laying hens and is resistant against numerous acaricides. Silver nanoparticles (AgNPs) represent an innovative solution against PRM. The current study aimed to assess the in vitro acaricidal activity of AgNPs against PRM and describe their potential mechanism of action. Nanoparticles were produced using a wet chemistry approach. Mites were collected using AviVet traps from 18 poultry farms in Greece. Contact toxicity bioassays were carried out for 24 h with negative controls, 20, 40, 60, or 80 ppm AgNPs. Analysis of variance was used to compare the mortality rates of PRM between the control and treatment groups, while LC_50_, LC_90_, and LC_99_ values were estimated using probit regression analysis for the total farms jointly and separately. Nanoparticles displayed strong acaricidal activity, and mortality rates were significantly different between groups and increased by AgNPs concentration. Overall mean LC_50_, LC_90_, and LC_99_ values were 26.5, 58.8, and 112.3 ppm, respectively. Scanning electron microscopy on mites treated with 80 ppm AgNPs revealed cracks in their exoskeleton and limb detachments, presumably resulting from the interaction between AgNPs and the mites’ chitin. Future studies should focus on assessing AgNPs residues in chicken tissues before moving into field trials.

## 1. Introduction

*Dermanyssus gallinae* (De Geer, 1778), also known as the poultry red mite (PRM), is the most common blood-sucking arthropod in the commercial laying hen industry [1]. Its haematophagous action stresses and irritates the hens, causing feather-pecking and anaemia, which in large infestations can prove fatal [2,3]. Simultaneously, egg production begins to drop while the weight, mass, and overall quality of eggs are reduced, thus leading to substantial production and financial losses [4]. Furthermore, *D. gallinae* can serve as a vector of pathogens with zoonotic potential, including bacteria and viruses such as *Salmonella* spp., *Erysipelothrix rhusiopathiae*, *Escherichia coli* and Influenza type A virus [5,6,7]. Regarding public health, numerous human infestation cases are reported either from hospitals where pigeons sometimes build their nest, poultry house personnel, or even from swallows at apartment windows [8,9,10]. In Europe, the prevalence of PRM in poultry farms is estimated at 83%, while in northern Greece, infestation prevalence reaches 100% [10,11]. Its life cycle comprises five distinct stages, with only nymphs and adults feeding on blood. PRM spends most of its time away from the host, hiding in cracks, metal connections, and crevices during the day. It comes out of its hiding spot at night and feeds on chickens for up to 60 minutes [1].

As a result, the control of PRM is usually based on spraying the laying hen facilities and equipment with acaricides instead of treating the hens directly [12]. In the past, the acaricidal treatment included the use of chemical compounds (organochlorines, organophosphates, carbamates, formamidines, synthetic pyrethroids, spinosyns, and phenylpyrazoles) [13,14,15,16]. Unfortunately, these compounds have been heavily misused, including but not limited to repeated use for many years, frequent use of the same acaricides, overdosing, and off-label use [2,17]. These malpractices have led to several compounds getting banned due to drug metabolite residues in eggs and hen tissues (i.e., meat) [17,18], removed from the market due to low efficacy/resistance reports [15,16] and environmental concerns [19]. Treatment of PRM is even more difficult, considering that mites can live up to 9 months without feeding and can quickly overpopulate in a matter of weeks when they become active again [20,21]. Environmental conditions, such as temperature and relative humidity, in modern commercial farms are ideal for the multiplication of PRM, whereas its control can be challenging due to its hiding nature and the high stocking density of layers in confined areas [3]. Currently, fluralaner is the only licensed product in Greece for treating hens with PRM infestations [15], and it is administered through drinking water. Nonetheless, it was only recently introduced in the market in 2017, and it is unknown yet if its extensive use will lead to a resistance of mites to this compound in the future. Other control measures include biological approaches (predatory mites, bacteria, entomopathogenic fungi, essential oils, plant extracts, and vaccines) [16,22,23,24,25]. However, even these methods have their drawbacks, i.e., essential oils exhibit variable acaricidal activity and are not trusted in the industry [26], while vaccines have low efficacy [27]. Lastly, physical control measures like inert dusts [28] have also been studied, but more research is required to validate their efficiency. Therefore, novel, alternative, and effective means to combat *D. gallinae* are urgently needed.

On the topic of alternative treatments, nanotechnology has gained more attention in both human and veterinary medicine in recent years due to the effectiveness of nanoparticles in a wide variety of biomedical applications and their future prospects. These include but are not limited to use as biosensors, diagnostic imaging techniques, targeted delivery of drugs, plant pathology and biotechnology, regenerative medicine, bioremediation and use against a vast array of pathogens [29,30]. The success of nanomaterials is primarily based on their extraordinary catalytic properties, high surface area-to-volume ratio, and excellent surface adsorption characteristics. Over the years, different metal nanoparticles have been manufactured with diverse techniques and materials, each having distinct benefits and drawbacks [29,30,31]. Silver nanoparticles (AgNPs) represent a novel and promising prospect to control PRM infestations without the risk of PRM developing resistance against them [32]. They could be employed by spraying the instruments and equipment of laying hen facilities (i.e., perches and under the egg conveyer belts) [17], including cracks and crevices where mites typically hide during the day. Moreover, AgNPs have been successfully used against different parasites, including helminths (nematodes, cestodes, and trematodes) [33,34,35], protozoa, such as *Toxoplasma gondii*, and *Leishmania* spp. [29,30,36,37] and even ectoparasites such as mosquitoes and ticks [38]. However, their exact mechanism of action remains unknown. Towards this end, the aim of the current study was to evaluate the contact toxicity of AgNPs at different concentrations against *D. gallinae* adults and elucidate their potential mechanisms of action.

## 2. Materials and Methods

### 2.1. Silver Nanoparticles Production

A wet chemistry approach was employed for the production of the AgNPs [39]; in brief: silver nitrate, purchased from Duchefa Biochemie (99.9% AgΝO3, Mr = 169.873 g/mol), was dissolved in deionized water and reduced to 1500 ppm of AgNPs by a reducing agent that was produced by components conventionally used in studies [40,41,42]. The suspension was stabilized with an aqueous solution containing a protein procured by Sigma Aldrich (20,000–25,000 g/mol) and a non-ionic surfactant purchased from Alfa Aesar (1000–2000 g/mol, with a purity of 98–99%). The colloidal suspension’s ratio of AgNPs and ions was adjusted through tangential flow filtration (TFF) with a 5 kDa membrane (Pall Corporation, New York, NY, USA). The silver content was subsequently evaluated through inductively coupled plasma—optical emission spectrometry (ICP-OES). 

### 2.2. Physicochemical Characterization

The size distribution profiles of the colloidal suspension were determined through dynamic light scattering (DLS) using a VASCO 3 DLS analyzer of Cordouan Technologies. The average particle size was verified by high-resolution transmission electron microscopy (HR-TEM), which provided additional information on the AgNPs’ morphology and shape (JEOL JEM 2010 & Oxford INCA). The UV-Vis spectra of the colloidal suspension were determined, upon a 10× dilution of the as-produced AgNPs, using a Cary 60 device (Agilent Technologies, Santa Clara, CA, USA). Finally, the zeta potential (surface charge) of the AgNPs was measured by a Laser Doppler Electrophoresis (LDE) technique using a Wallis Zeta analyzer (Cordouan Technologies, Bordeaux, France).

### 2.3. Mite Collection and Identification

Live *D. gallinae* mites were collected using special cardboard traps, AviVet Red Mite Trap™ (Avivet, adVee Dierenartsen, Heeswijk Dinther, The Netherlands) [43], from 18 commercial and backyard laying hen farms located in different regions of Greece during the summer of 2022. There was no acaricide application/treatment in any of the 18 farms during the last three months before placing the traps. After one week, the traps were collected, sealed inside plastic bags, and returned to the Laboratory of Parasitology and Parasitic Diseases, School of Veterinary Medicine, Faculty of Health Sciences, Aristotle University of Thessaloniki, Greece. Mites from all 18 laying hen farms were identified as *D. gallinae* after examination under a stereomicroscope (Olympus, Research Stereomicroscope System SZH10) and based on morphological keys [44]. Traps were kept inside the sealed plastic bags at 23 ± 1 °C until bioassays began.

### 2.4. Contact Toxicity Bioassays

All 18 different bioassays with AgNPs were performed within two days after the traps had arrived at the laboratory. Before starting the bioassays, the traps were placed inside the freezer (−20 °C) for 15 min to reduce mite mobility and make them easier to handle. Whatman Grade 1 filter paper (5.5 cm diameter) was placed at the bottom of plastic Petri dishes (6 cm diameter). Each paper was sprayed 1 time either with 20, 40, 60, or 80 ppm AgNPs, while negative controls with deionized water were also used. The containers used for spraying were identical, and the papers were sprayed from a distance of 15 cm. For the bioassays, the mites were separated into groups of 10 PRM adults using a fine brush and placed inside the Petri dishes. After placing them inside the Petri dishes, the mites were sprayed 1 time with AgNPs/distilled water of the same concentration (ppm) as the paper before closing the lid of each dish. All Petri dishes were placed inside a large plastic container, and the container edges were covered with petroleum jelly to prevent mites from escaping. A total of 3 biological replicates were performed concurrently for each concentration, and the total volume of AgNPs/distilled water per Petri dish was 0.5 mL (2 squirts), ensuring that the whole filter paper was covered. A total of approximately 120 mites were used for each bioassay. Spraying was done from the lowest to the highest concentrations of AgNPs, and separate disposable gloves were used for each concentration to minimize the risk of AgNPs carrying over between concentrations. After 24 h, the mites were checked to assess how many were still alive and how many were dead (mortality) under the same binocular stereomicroscope used to identify them. According to the published methodology on *D. gallinae* bioassays [12,45], the mites were considered alive if they showed any form of repetitive movement, either spontaneously or in response to a gentle touch with a fine brush. For negative controls, natural mortality close to 10% was considered ideal. The mortality rate for each unique replicate was calculated by dividing the number of dead mites by the number of total mites used in that specific Petri dish. All bioassays were performed at a steady temperature of 23 ± 1 °C and a relative humidity of 70 ± 5% under 14:10 lighting conditions (light/dark). The same experienced person performed all bioassays to avoid any unintended bias.

### 2.5. Scanning Electron Microscopy

To elucidate the potential mechanism of action of AgNPs against PRM, live mites were separated into 3 different groups. Group A comprised mites killed via freezing inside the freezer (−20 °C) for 1 day. Group B consisted of mites drowned in Ethanol 99% for 1 day. Finally, Group C contained mites killed in the bioassays with AgNPs at 80 ppm concentration. Groups A and B were used as controls to establish a baseline for comparing external morphological differences with dead mites from Group C. All the mites from the three groups were considered dead if they showed no movement, either spontaneously or in response to a gentle touch with a fine brush. Consequently, mites from the three different groups were examined under a scanning electron microscope (SEM) (JSM-IT500, JEOL Ltd., Tokyo, Japan) to determine any morphological differences among them.

### 2.6. Statistical Analysis

Analysis of variance (ANOVA) was used to compare the mortality rates of *D. gallinae* between the control group (0 ppm AgNPs) and the four treatment groups (20, 40, 60, and 80 ppm AgNPs); Tukey HSD was used as a post-hoc test. 

To measure the relationship between AgNPs concentration and the proportion of *D. gallinae* that died within 24 h, the lethal concentration that killed 50% (LC_50_), lethal concentration that killed 90% (LC_90_), and lethal concentration that killed 99% (LC_99_) values and their 95% confidence intervals were estimated using probit regression analysis for the total of the farms jointly and separately; in both cases, Pearson goodness-of-fit was estimated to assess whether the models fitted the data well. For the statistical analyses, SPSS v23 was used, and statistical significance was set at the a = 0.05 level.

Finally, the interpretation of acaricidal activity was based on the work of Kim et al. [16], as shown:

- mortality >80% → strong,
- mortality 80–61% → moderate,
- mortality 60–40% → weak, and
- mortality <40% → little or no activity

## 3. Results

### 3.1. Physicochemical Characterization

**Figure 1:**
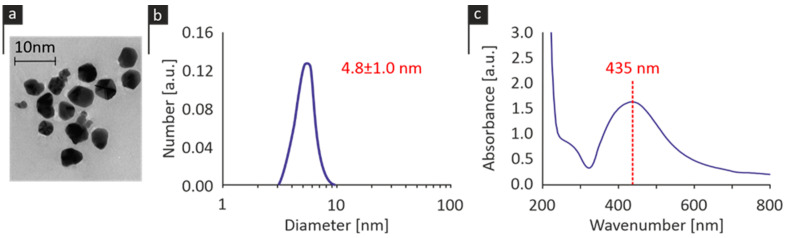
(**a**) Particle size and shape as determined by HR-TEM, (**b**) size distribution of the NPs populations, and (**c**) UV-Vis spectrum of the AgNPs sample.

The HR-TEM revealed that the AgNPs were of a spherical shape, with an average size of approximately 5 nm, as illustrated in Figure 1a. This size distribution was verified by the DLS measurements, which indicated a monodispersed population of AgNPs with an average size of 4.8 ± 1.0 nm (Figure 1b). The UV-Vis spectroscopy exhibited a wavelength consistent with silver nanoparticles, i.e., at an absorption peak of 435 nm, as demonstrated in Figure 1c. The silver content, as evaluated by ICP-OES, revealed a 1:1 % allocation of AgNPs and silver ions, respectively, while AgNPs had a ζ-potential (surface charge) of 14.8 mV.

### 3.2. Bioassays Results

**Figure 2:**
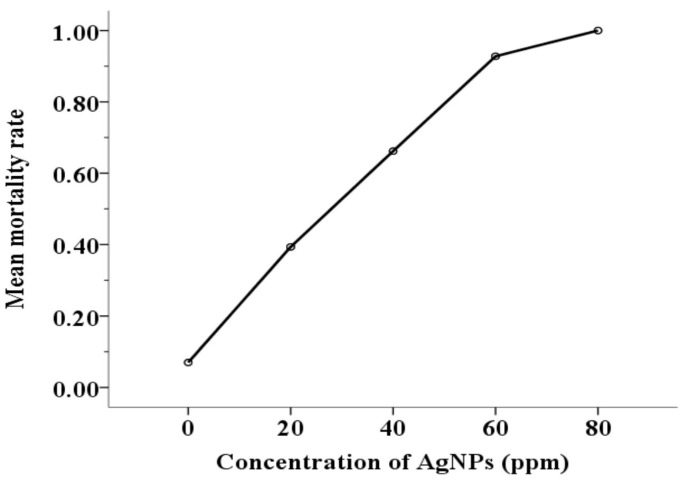
Mean mortality rates for the control and four treatment groups (20, 40, 60, and 80 ppm AgNPs).

The mean values of mortality rates are presented in Figure 2. Additionally, the mean mortality rates for the control and four treatment groups (20, 40, 60, and 80 ppm AgNPs), as well as the classification of acaricidal activity proposed by Kim et al. [16], are presented in Table 1. Mortality rates were significantly different between groups [F(4, 211) = 619.7, *p* < 0.001]. In particular, in all cases, mortality rates were significantly higher in the treated groups compared to the control group at the 0.001 level. Partial comparisons between the groups are summarized in Table 2.

Pearson goodness-of-fit tests indicated that the models adequately fitted the data either whether farms were jointly [*X*^2^(df = 160) =148.42, *p* = 0.734] or separately [*X*^2^(df = 143) =112.43, *p* = 0.972] considered. Overall, mean values of AgNPs LC_50_, LC_90,_ and LC_99_ are presented in Table 3 and were 26.5, 58.8, and 112.3 ppm in the studied farms, while the respective values varied from 20.4 to 32.4, 42.8 to 68.0, and 78.3 to 124.5 ppm among farms.

### 3.3. Scanning Electron Microscopy Results

**Figure 3:**
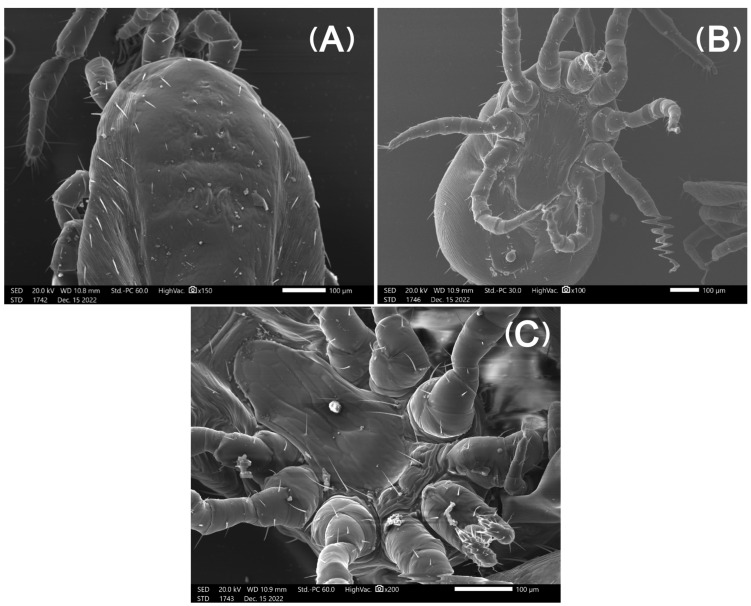
Micrographs of *Dermanyssus gallinae* after freezing them at (−20 °C) for 1 day: dorsal (**A**) and ventral (**B**,**C**) views. Exoskeletons and limbs are intact with no visual deformities.

**Figure 4:**
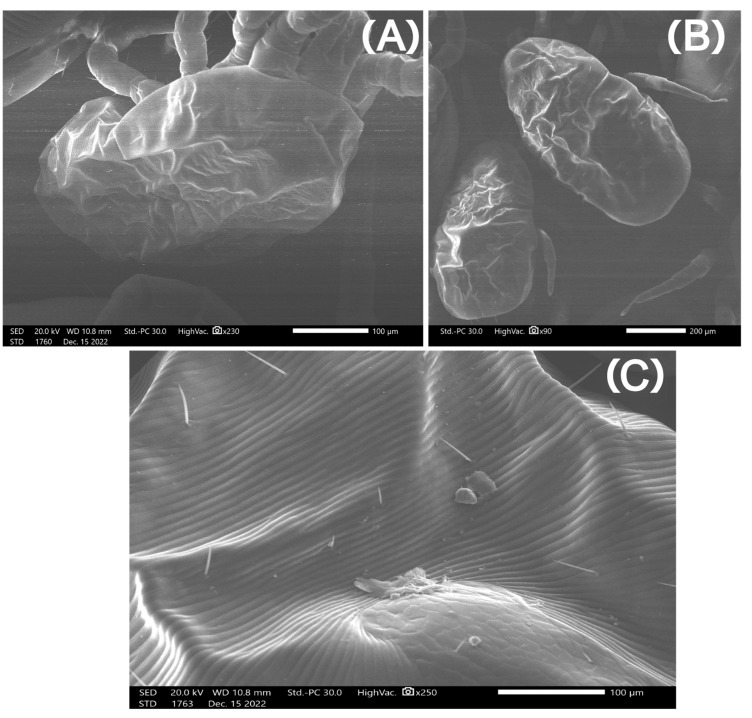
Micrographs of *Dermanyssus gallinae* after placing them in 99% ethanol for 1 day. (**A**–**C**) There is a loss of external morphological characteristics, severe dehydration, and large indentations in the exoskeleton.

**Figure 5:**
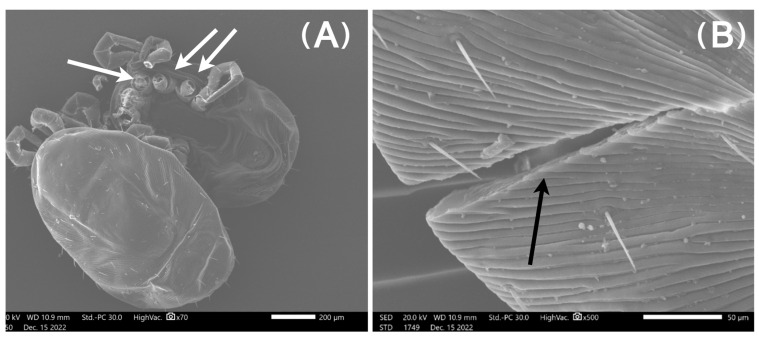
Micrographs of *Dermanyssus gallinae* after bioassays with AgNPs at 80 ppm for 1 day: (**A**) There are visible leg detachments (white arrows) and (**B**) considerable cracks throughout their exoskeleton (black arrow).

**Figure 6:**
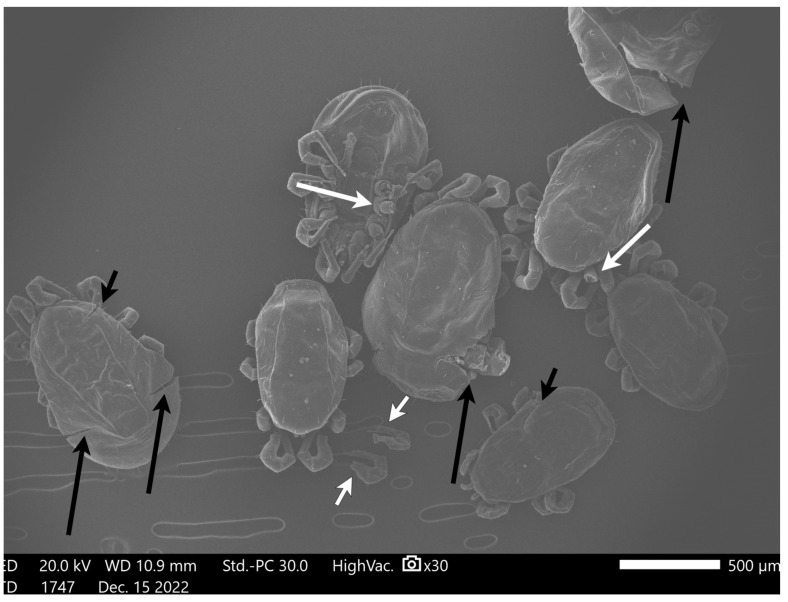
Micrograph of *Dermanyssus gallinae* after bioassays with AgNPs at 80 ppm for 1 day. There are numerous visible leg detachments (white arrows) and considerable cracks throughout their exoskeleton (black arrows).

Micrographs from mites in Group A (freezing) captured with the SEM showed mites with intact exoskeletons and no visual deformities (Figure 3). In contrast, in mites from Group B (ethanol), there was a loss of external morphological characteristics, severe dehydration, and large indentations in the exoskeleton (Figure 4). Finally, mites from Group C (AgNPs 80 ppm) exhibited considerable cracks throughout their exoskeleton and leg detachments (Figure 5 and Figure 6). 

## 4. Discussion

The fundamental idea behind nanomedicine is the meticulous engineering of nanoparticles that allows for the use of fewer chemical compounds at lower dosages to ensure better treatment outcomes [32]. According to in vitro bioassays with AgNPs against ticks and mosquitos and bioassays with acaricides against PRM, the 24-h exposure time was chosen as the most appropriate duration for the current bioassays [38,46]. The mean natural mortality rate using deionized water was 7 ± 1.3%, and ideal since it was less than 10%. At 20 ppm concentration (mean mortality rate = 39 ± 2.7%), AgNPs had low acaricidal activity. The nanoparticles at 40 ppm (mean mortality rate = 66 ± 1.9%) displayed moderate acaricidal activity. Finally, AgNPs exhibited strong acaricidal activity against PRM adults (mortality >80%) [16] at concentrations of 60 ppm (mean mortality rate = 93 ± 6.4%) and 80 ppm (mean mortality rate = 100 ± 0%). 

Regarding the overall LC_50_ value, AgNPs achieved the death of 50% of mites at a quite low concentration of only 26.5 ppm. The overall LC_90_ and LC_99_ values were higher (58.8 ppm and 112.3 ppm, respectively), but they are on the very low end of the spectrum of effective acaricidal concentrations compared with other compounds [15,47,48,49]. Per farm, LC_99_ values (78.3–124.5 ppm) ranged more than the respective LC_90_ values (42.8–68.0 ppm) but still were not highly different in contrast to other LC_90_ values of PRM bioassays with different field populations [15]. Specifically, when compared with other in vitro contact toxicity bioassays, the overall mean value of AgNPs LC_90_ = 58.8 ppm (ranging per farm from 42.8–68.0 ppm) was comparable to the per farm LC_90_ = 15.6–62.5 ppm of fluralaner against a German laboratory isolate of PRM [15]. In one case, it was lower than the LC_90_ = 125 ppm of fluralaner against a Brazilian field isolate. Furthermore, the per-farm LC_90_ values of AgNPs against all field isolates of PRM in the current study were substantially lower than the LC_90_ of deltamethrin (>1000 ppm), cypermethrin (>1000 ppm), phoxim (>4000 ppm), propoxur (>1000 ppm), and spinosad (>4000 ppm) against other specific field isolates [15]. Although the exact methods used for contact toxicity bioassays are different between the present study and the one by Thomas et al. [15], we examined more PRM field isolates (18 compared to 13), and none were resistant to AgNPs. This finding is in agreement with other studies showing that nanoparticles do not induce resistance in parasites [32], and to the best of the authors’ knowledge, there are no resistance reports of parasites against AgNPs. 

Concerning AgNPs’ potential mechanism of action, the authors of the current study propose that there was an uptake of AgNPs inside the bodies of PRM that led to their death. Before analyzing the exact mechanism, it is essential to understand a few key concepts about the mite’s anatomy and biology. Mites have an exoskeleton that contains chitin, and in adults, this chitin creates a sclerotized cuticle layer that is rigid, outlines their body structure, and helps with muscle connection [19]. Inside their bodies, PRM have a digestive system containing a peritrophic membrane that also consists of chitin and aids blood digestion [19,50]. The inhibition or binding of chitin in this membrane has been successfully employed to control other mite species [19], and in PRM, the use of the chitin-inhibitor triflumuron under field conditions caused a mite reduction of more than 70% for five months [51]. Therefore, treatments targeting chitin represent a feasible and safe solution for combating the PRM since chickens and mammals lack chitin [19]. Based on this information, *D. gallinae* either absorbed AgNPs inside its body through its pores, inhalation with its spiracle and tracheal structures, or directly absorbed them into its chitin exoskeleton. In the latter case, the absorption of AgNPs may be attributed to two different paths: i) via electrostatic interactions of chitin and its functional groups with the positively charged AgNPs [52], or ii) the strong affinity of chitin toward AgNPs, mediated by the polymer’s acetamino groups [53]. Regardless, however, of the absorption route, the immobilization of AgNPs within the arthropod’s exoskeleton or its peritrophic membrane is expected to increase its brittleness since nanoparticles cause dislocations decreasing the mobility of the polymer’s chains [54]. In a recent study, Liu et al. (2014) argued in favor of the chitin-mediated uptake of AgNPs, showing that crab-shell-derived chitin powder absorbs AgNPs at rates as high as 19.8 mg/g [52], while literature data affirm a notable increase in stiffness of nano-doped chitin when compared to its pure counterpart [55,56].

This mode of action is in good agreement with the SEM micrographs presented here, as the arthropods treated with AgNPs exhibited considerable cracks throughout their exoskeleton. Although the PRM’s normally ductile shells are expected to absorb AgNPs only at their most superficial layer, the formation and subsequent acceleration of cracks in this surface, followed by the suppression of multiple crazing in the pure chitin substrate, ultimately results in the shell’s brittle fracture [57]. This surface embrittlement was also evident in the PRM extremities, where chitin helps with attachment and is likely the aetiology for the detachment of their limbs. The interaction between AgNPs and chitin in the parasites’ cuticle has also been observed in nematodes, where nanoparticles exhibited nematocidal activity [58]. The nanoparticles’ mechanism of action was not through dehydration of treated mites. On the other hand, ethanol caused dehydration and large indentations in mites, as showcased in previous studies [59].

Another point to consider is the safety of using AgNPs in the context of One Health (animals, humans, and the environment). In general, nanoparticles are considered non-toxic, but that heavily depends on their synthesis and physiochemical characteristics like size, structure, and electric charge [32]. Smaller nanoparticles and those with a higher electric charge, like the ones synthesized in the current study (average size: 4.8 ± 1.0 nm and surface charge: +14.8 mV), are considered safer [32]. Nanoparticles should be quickly eliminated from hens, and any residues in eggs, tissues, and organs should be measured to ensure they are lower than the major residue limits (MRLs) so that animal welfare and consumer safety are protected. As such, in vivo toxicity trials in laying hens are required using AgNPs with the same shape, physicochemical characteristics, and size as the ones employed in the current study. Consequently, the efficacy of AgNPs against PRM should be demonstrated under field conditions, with the authors of the current study suggesting a concentration near the upper limit of the individual LC_99_ values (i.e., 124.5 ppm as in Farm 8 and Farm 18).

## 5. Conclusions

Silver nanoparticles constitute a novel and promising solution in the fight against *D. gallinae* that is effective and without the risk of developing resistance, and they can assist any other control method. In the bioassays with PRM from all 18 farms tested, AgNPs displayed strong acaricidal activity at 60 and 80 ppm concentrations. Overall LC_90_ and LC_99_ values were low and comparable with other acaricides used in today’s market. Scanning electron microscopy micrographs of PRM treated with AgNPs at 80 ppm revealed considerable cracks in their chitin exoskeleton and leg detachments. The potential mechanism of action suggested was the interaction of AgNPs with chitin in the mite’s exoskeleton or peritrophic membrane. Further studies are required to assess AgNPs’ residues in chicken tissues before moving into field trials.

## Figures and Tables

**Table 1:** Mean mortality rates for the control and the four treatment groups (20, 40, 60, and 80 ppm AgNPs) after 24 h and classification of acaricidal activity.

Concentration of AgNPs (ppm)	Mean Mortality Rate ± S.E.	Classification of Acaricidal Activity
0	0.07 ± 0.013	-
20	0.39 ± 0.027	Little or no activity
40	0.66 ± 0.019	Moderate
60	0.93 ± 0.064	Strong
80	1.00 ± 0.00	Strong

S.E.: Standard error.

**Table 2:** Partial comparisons of mortality rates between the control and the four treatment groups (20, 40, 60, and 80 ppm AgNPs).

Treatment I	Treatment J	Mean Difference (I − J)	S.E.	*p*-Value	95% Confidence Interval	
					Lower Bound	Upper Bound
0 ppm	20 ppm	−0.32	0.024	0.000	−0.39	−0.26
	40 ppm	−0.59	0.020	0.000	−0.65	−0.54
	60 ppm	−0.86	0.020	0.000	−0.91	−0.80
	80 ppm	−0.93	0.024	0.000	−1.00	−0.86
20 ppm	40 ppm	−0.27	0.024	0.000	−0.34	−0.20
	60 ppm	−0.53	0.024	0.000	−0.60	−0.47
	80 ppm	−0.61	0.028	0.000	−0.68	−0.53
40 ppm	60 ppm	−0.27	0.020	0.000	−0.32	−0.21
	80 ppm	−0.34	0.024	0.000	−0.41	−0.27
60 ppm	80 ppm	−0.07	0.024	0.028	−0.14	−0.01

S.E.: Standard error.

**Table 3:** Overall and per farm LC_50_, LC_90_, and LC_99_ values and their 95% confidence intervals (ppm).

	LC_50_ (95% CI)	LC_90_ (95% CI)	LC_99_ (95% CI)
Farm 1	24.5 (21.2–28.1)	51.4 (44.6–60.0)	94.1 (79.2–115.0)
Farm 2	29.1 (24.9–33.9)	61.1 (52.0–73.1)	111.9 (92.2–140.4)
Farm 3	21.2 (17.6–25.2)	44.4 (37.4–53.3)	81.4 (67.1–101.2)
Farm 4	20.4 (16.3–25.1)	42.8 (34.7–53.1)	78.3 (62.6–100.2)
Farm 5	23.9 (19.4–29.3)	50.2 (41.0–62.4)	92.0 (73.5–118.4)
Farm 6	25.2 (20.9–30.1)	52.9 (44.0–64.3)	96. 8 (78.7–122.5)
Farm 7	31.2 (26.1–37.1)	65.5 (55.0–79.1)	119.9 (98.3–150.7)
Farm 8	32.4 (27.9–37.5)	68.0 (58.2–80.8)	124.5 (103.1–155.3)
Farm 9	29.3 (24.4–35.1)	61.5 (50.9–75.5)	112.6 (90.7–144.4)
Farm 10	27.1 (21.6–33.5)	57.0 (46.3–70.2)	104.3 (84.2–131.6)
Farm 11	29.6 (23.6–36.5)	62.1 (50.6–76.5)	113.7 (91.8–143.7)
Farm 12	26.2 (21.0–32.2)	55.1 (45.1–67.4)	100.8 (82.0–126.3)
Farm 13	26.2 (20.8–32.5)	55.0 (44.6–68.2)	100.7 (80.9–127.8)
Farm 14	27.3 (21.8–33.7)	57.3 (46.7–70.7)	104.9 (84.7–132.7)
Farm 15	31.7 (26.1–37.9)	66.5 (55.7–80.0)	121.7 (100.3–151.3)
Farm 16	27.9 (22.3–34.4)	58.6 (47.7–72.2)	107.2 (86.6–135.5)
Farm 17	30.8 (25.0–37.4)	64.7 (53.5–78.7)	118.4 (96.7–148.3)
Farm 18	32.4 (26.6–38.9)	68.0 (56.7–82.2)	124.5 (102.1–155.3)
Overall	26.5 (24.8–28.1)	58.8 (55.5–62.8)	112.3 (100.9–128.1)

## Data Availability

Not applicable.
